# Identifying alcohol misuse biotypes from neural connectivity markers and concurrent genetic associations

**DOI:** 10.1038/s41398-022-01983-1

**Published:** 2022-06-16

**Authors:** Tan Zhu, Chloe Becquey, Yu Chen, Carl W. Lejuez, Chiang-Shan R. Li, Jinbo Bi

**Affiliations:** 1 Department of Computer Science and Engineering, School of Engineering, University of Connecticut, Storrs, CT USA; 2 Department of Psychiatry, School of Medicine, Yale University, New Haven, CT USA; 3 Department of Psychological Sciences, College of Liberal Arts and Sciences, University of Connecticut, Storrs, CT USA; 4 Department of Neuroscience, School of Medicine, Yale University, New Haven, CT USA; 5 Wu Tsai Institute, Yale University, New Haven, CT USA

**Keywords:** Diseases, Biomarkers

## Abstract

Alcohol use behaviors are highly heterogeneous, posing significant challenges to etiologic research of alcohol use disorder (AUD). Magnetic resonance imaging (MRI) provides intermediate endophenotypes in characterizing problem alcohol use and assessing the genetic architecture of addictive behavior. We used connectivity features derived from resting state functional MRI to subtype alcohol misuse (AM) behavior. With a machine learning pipeline of feature selection, dimension reduction, clustering, and classification we identified three AM biotypes—mild, comorbid, and moderate AM biotypes (MIA, COA, and MOA)—from a Human Connectome Project (HCP) discovery sample (194 drinkers). The three groups and controls (397 non-drinkers) demonstrated significant differences in alcohol use frequency during the heaviest 12-month drinking period (MOA > MIA; COA > non-drinkers) and were distinguished by connectivity features involving the frontal, parietal, subcortical and default mode networks. Further, COA relative to MIA, MOA and controls endorsed significantly higher scores in antisocial personality. A genetic association study identified that an alcohol use and antisocial behavior related variant rs16930842 from LINC01414 was significantly associated with COA. Using a replication HCP sample (28 drinkers and 46 non-drinkers), we found that subtyping helped in classifying AM from controls (area under the curve or AUC = 0.70, *P* < 0.005) in comparison to classifiers without subtyping (AUC = 0.60, not significant) and successfully reproduced the genetic association. Together, the results suggest functional connectivities as important features in classifying AM subgroups and the utility of reducing the heterogeneity in connectivity features among AM subgroups in advancing the research of etiological neural markers of AUD.

## Introduction

Alcohol use disorder (AUD) is a major public health problem in the United States and worldwide [1]. AUD is characterized by a variety of behavioral criteria, as described in the Diagnostic and Statistical Manual of Mental Disorders (DSM) [2], posing significant challenges to etiologic research [3–5]. Understanding the different dimensions of alcohol use behavior and their neural and genetic basis would facilitate the development of new treatment and prevention strategies for AUD. Etiological research of AUD has focused on the neurobiology of reward processing, cognitive control and emotion regulation [6, 7]. Adoption, twin, and family studies show that the behavioral and neural phenotypes of AUD and problem drinking are influenced by genetics [8–12]. It is essential to characterize the genetic, neural, and behavioral determinants to differentiate the biotypes of alcohol misuse for precision medicine. This study aims to utilize the Human Connectome Project (HCP) [13] data to identify distinct biotypes of alcohol misuse.

Biological, clinical, and analytical variances embedded in alcohol use-related phenotypes reduce statistical power and diminish evidence of clinically impactful classification. Clinical symptoms, such as the DSM criteria, are often used in the identification of alcohol use subtypes. However, they represent distal behavioral manifestations, and the resultant subcategories may not effectively distinguish etiological subtypes and help in identifying genetic risk variants [14]. Magnetic resonance imaging (MRI) quantifies structural and functional brain differences that dispose individuals to and/or reflect the effects of alcohol use. Thus, MRI provides intermediate endophenotypes of problem alcohol use, such as binge drinking, and helps in assessing the genetic architecture of addiction or alcohol use behavior. There is a long tradition of typological research in psychiatry that has helped in refining the DSM criteria of substance use disorders. Whereas early topology research employed univariate classification based on a single factor (e.g., Type I and Type II AUD distinguished by age of onset [15, 16]), multivariate subtyping on the basis of a variety of clinical features reflecting vulnerability, severity, chronicity, and psychopathology has been shown to be more effective [17, 18]. However, empirical subtyping by symptoms has not yielded clinically impactful categories [19], and it has been proposed that neural markers may enhance the validity in subtyping alcohol misuse [20, 21]. For instance, using unique and stable connectivity patterns characterized by resting-state functional MRI (rsFMRI), investigators have defined four subtypes of depression with differential responses to treatment [22]. Further, these biological subtypes may be underpinned by different genetic risk factors. Thus, this study attempted to employ statistical methods to link brain circuits and genetic markers for sub-categorization of drinking behavior so to enhance the validity of clinically derived patterns.

In research of the connectivity markers of alcohol misuse, investigators can examine the whole brain or specific neural circuits. For instance, alcohol misuse appears to disproportionately affect the thalamus and hippocampus [23–34]. We characterized earlier how thalamic connectivities were associated with alcohol expectancy and the extent of problem drinking in non-dependent drinkers [33]. Other studies have described the effects of short- and long-term alcohol use on whole-brain connectivity and associated the connectivity patterns with clinical manifestations of AUD [35, 36] and identified connectivity markers that improve the prediction of AUD diagnosis [37–39].

We employed whole-brain functional connectivity features (FCs) of the HCP data derived from rsFMRI to achieve two aims. First, we employed connectivity features to identify biotypes of alcohol misuse (AM). We hypothesized that subtyping would help in improving the classification, as compared to the analysis using the same but non-differentiated subjects, of drinkers vs. non-drinkers. Second, we aimed to characterize the clinical features and genetic risk variants of the biotypes and hypothesized that the AM biotypes would be distinguished by clinical features and risk variants that would otherwise not reveal by merely comparing drinkers with non-drinkers.

## Subjects and methods

### Subjects

We used the HCP S1200 Subjects Release data of 1206 young adults (age 22–35), where 1033 were genotyped and underwent 3 T rsFMRI scans twice (scan 1 and scan 2). Although the HCP project aimed at studying neurotypical populations, many subjects used substances of abuse, particularly alcohol, tobacco, and marijuana, meeting at least one DSM-IV criterion of AUD, nicotine use disorder (NUD), or marijuana use disorder (MUD).

Our study targeted the subjects meeting at least one of the diagnostic items of AUD, hereafter termed subjects of alcohol misuse (AM). After preprocessing of the rsFMRI images [40]. (Section 2.3), 60 of the 1033 subjects were excluded because of poor image quality and/or failed registration. We further excluded those who met at least one diagnostic items of NUD or MUD and those without genotype data. As a result, 739 subjects were included in the final sample for analyses. Among the 739, 250 were AM subjects (132 men; 22–35 with mean ± SD = 28.3 ± 3.6 years) and 489 control subjects (189 men; 22–36 with mean ± SD = 28.8 ± 3.8 years). Of the 250 AM subjects, 106 met criteria for AUD (i.e., abuse or dependence) [41] and 68 (including 43 with AUD) engaged in binge-drinking (having ≥ 4/5 drinks per occasion for women/men) at least once a week in the past 12 months. The detailed demographic characteristics of AM subjects and controls are summarized in Supplementary Table S1.

### Clinical measures

In the HCP alcohol-related variables were assessed through the administration of the Semi-Structured Assessment for the Genetics of Alcoholism (SSAGA), a computer-assisted interview that yields lifetime DSM-IV diagnosis of substance user disorders [42]. Ten variables of numerical values quantified the frequency and quantity of alcohol use (Supplementary Table S2). These measures were linearly normalized into a scale of 0 to 5 to reflect the severity of drinking. The HCP also characterized psychiatric symptoms by the Adult Self Report (ASR) score [43] in the DSM-oriented scales, with DSM raw scores for depression, anxiety, severity of somatic symptoms, avoidant personality, attention deficit hyperactivity disorder (ADHD, including inattention and hyperactivity/impulsivity subscales), and antisocial personality.

### Functional connectivity features

All imaging data were acquired on a customized Siemens 3 T Skyra with a standard 32-channel Siemens receiver head coil and a body transmission coil. T1-weighted high-resolution structural images were acquired using a 3D MPRAGE sequence with 0.7 mm isotropic resolution (FOV = 224 mm, matrix = 320, 256 sagittal slices, TR = 2400 ms, TE = 2.14 ms, TI = 1000 ms, FA = 8°) and used to register rsFMRI data to a standard brain space. The rsFMRI data were collected in two sessions, using gradient-echo echo-planar imaging (EPI) with 2.0 mm isotropic resolution (FOV = 208 × 180 mm, matrix = 104 × 90, 72 slices, TR = 720 ms, TE = 33.1 ms, FA = 52°, multi-band factor = 8). Within each session, oblique axial acquisitions alternated between phase encoding in a right-to-left (RL) direction in one run and phase encoding in a left-to-right (LR) direction in the other run. Each run lasted 14.4 minutes (1200 frames). Physiological data (i.e., cardiac and respiratory signals) were also acquired, using a standard Siemens pulse oximeter placed on a digit and a respiratory belt on the abdomen, and sampled equally at 400 Hz (~288 samples per frame). More details of the data collection procedures can be found in the HCP S1200 Release Reference Manual.

In the current study, both sessions (LR and RL runs combined for each session) of rsFMRI data were used and processed with Statistical Parametric Mapping (SPM12, Wellcome Department of Imaging Neuroscience, University College London, U.K.). Images of each participant were first realigned (motion corrected) and a mean functional image volume was constructed from the realigned image volumes. These mean images were co-registered with the high-resolution structural MPRAGE image and segmented for normalization with affine registration followed by nonlinear transformation. The normalization parameters determined for the structural volume were then applied to the corresponding functional image volumes for each participant. Afterwards, the images were smoothed with a Gaussian kernel of 4 mm at Full Width at Half Maximum.

Physiological signals were regressed out to reduce spurious BOLD variances. A temporal band-pass filter (0.009 Hz < f < 0.08 Hz) was also applied to the time course to obtain low-frequency fluctuations [44]. Further, we applied a “scrubbing” method to eliminate global motion-related artifacts. Specifically, frame-wise displacement FD(t) = |Δdx(t)| + |Δdy(t)| + |Δdz(t)| + |Δα(t)| + |Δβ(t)| + |Δγ(t)| was computed for every time point t, where (dx, dy, dz) and (α, β, γ) are the translational and rotational movements, respectively [45]. Moreover, the root mean square variance of the differences (DVARS) in % BOLD intensity I(t) between consecutive time points across brain voxels, was computed – DVARS(t) = sqrt(|I(t) – I(t-1)|2) – where the brackets indicated the mean across brain voxels. Following previous HCP studies, we marked volumes with FD > 0.2 mm or DVARS > 75 as well as one frame before and two frames after these volumes as outliers (censored frames). Uncensored segments of data lasting fewer than five contiguous volumes were also labeled as censored frames [46]. From each rsFMRI image, only uncensored frames were used in the computation of the correlation matrix of 268 regions of interest (ROIs) [47], which yielded 35,778 unique FC markers.

### Genotypes

The genotype data of all 739 subjects were obtained from the Database of Genotypes and Phenotypes (dbGaP) website [48], under phs001364.v1.p1. In this dataset, DNA samples were extracted from blood or saliva and genotyped with a custom microarray chip consisting of the Illumina Mega Chip, ImmunoArray, and psychiatry-related content from the PsychArray. Samples were also processed with a second Illumina Neuro Consortium chip for SNPs particularly relevant to neuroimaging studies [49]. Although genome-wide single nucleotide polymorphisms (SNPs) were available for the current 739 subjects, the HCP sample size is not adequately powered for a genome-wide association study (GWAS). Thus, we performed candidate gene analysis by selecting the genetic variants from the genotyped SNPs previously associated with AUD as summarized in the NHGRI-EBI GWAS Catalog [50]. This mapping with quality control resulted in 3890 SNPs in 75 AUD-related genes (Supplementary Table S3).

### Machine learning and classification

#### Overall analytic goals and routines

Of the 739 subjects, 665 (222 AM subjects) and 74 (28 AM) were used for discovery and replication, respectively. The discovery set was further split into a training (*n* = 591, 194 AM) and validation (*n* = 74, 28 AM) set so the classifiers can be validated without involving the replication set. The training, validation, and replication samples had matched proportions of AM subjects, demographic and psychiatric characteristics, and alcohol use behavior (Supplementary Table S4).

**Fig. 1:**
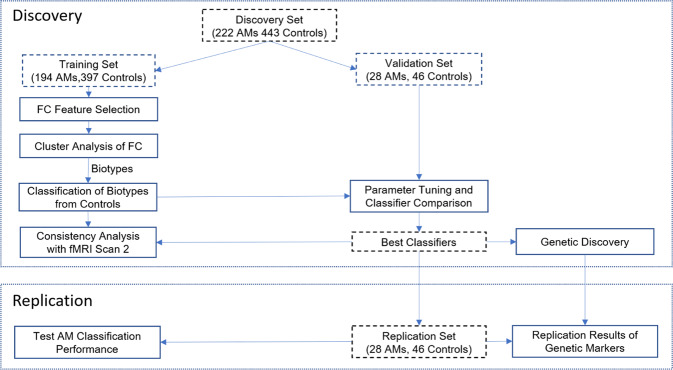
The analytic pipeline to identify and validate alcohol misuse (AM) biotypes.

Figure 1 shows the analytic procedures. We accounted for the covariate effects [51, 52] in the computation of each FC feature by regressing out age, sex and head motion in a linear model of the training data. We used the total number of censored frames to measure the level of head motion. Other datasets were corrected using the same linear models of training data. The training sample was used to identify AM biotypes with selected FC features and to create classifiers for AM biotypes. The validation sample was used to compare classifiers and select the best according to the classification accuracy of AM vs. control subjects. The resultant classifiers produced quantitative scores for each subject in terms of membership of a specific biotype, which also served as quantitative traits in genetic association tests. During replication, the validated classifiers were applied to replication sample to report classification accuracy. Genetic markers identified during discovery were tested for replication, too.

#### Feature selection and biotype identification

We performed feature selection, dimension reduction, and cluster analysis. First, we searched for top FC features that were significantly Spearman’s rank-correlated with one or more of the ten alcohol use metrics and eight Adult Self Report (ASR) scores as described earlier. We then identified the top FC features that differed significantly between AM and control subjects in Wilcoxon rank-sum tests. We took a union of these features for cluster analysis. In this step, we set the significance threshold as 0.05, 0.005 and 0.0005 to identify three sets of FC features, and, subsequently, selected the set with the best AM biotyping performance.

We reduced the data dimension by the Uniform Manifold Approximation and Projection (UMAP), a novel nonlinear dimension reduction and visualization technique [53]. UMAP preserves the subject proximity in the sample when searching for a low dimensional space to map high dimensional data. We used UMAP to compress each of the three identified sets of FC features into 2, 3, and 4 dimensions, respectively, which resulted in 9 distinct UMAP representations. For each of the 9 representations, K-means and hierarchical clustering were used to derive AM clusters, with the number of clusters (2, 3 and 4) determined by the Variance Ratio Criterion, the ratio of the sum of between-clusters dispersion and of inter-cluster dispersion for all clusters. We trained the AM classifiers with these 9 AM biotype assignment solutions and selected the solution with the best classification performance. We then examined the resultant clusters in terms of the demographic, psychiatric, alcohol use, and rsFMRI-related characteristics.

#### Classifying AM biotypes from controls

AM biotyping helps reduce between-subject heterogeneity in alcohol-related FC features so to facilitate the classification of AM subjects. We constructed an artificial neural network (ANN) to discriminate AM subjects from controls by first mapping them to the biotypes (workflow shown in Supplementary Fig. S1). The resultant classifier could thus be used to classify replication subjects in terms of AM versus control. The input to the ANN is a whole-brain FC matrix containing 35778 distinct correlations pair-wise of the 268 ROIs. To reduce the number of trainable parameters in the ANN and render the model less prone to overfitting, the ANN performed feature extraction to map the high dimensional whole-brain FC matrix into a low dimensional representation (details in Supplementary Methods).

ANN models were trained in two separate procedures: to directly classify AM subjects from controls (the baseline ANN model); or to first distinguish biotypes from controls and then combine the biotype scores in a weighted sum to arrive the final AM prediction (i.e., ANN model with biotype knowledge). This strategy could compare whether biotyping improves the AM classification. In the second procedure, the ANN model was trained with a newly designed objective function which minimized the biotype classification error together with the overall AM versus control classification error (details in Supplementary Methods). By comparing the area under the receiver operating characteristic curve (AUC) of the resultant classifiers on the validation sample, we selected the best clustering solution and corresponding classifiers. The validation set was also used to determine the threshold to convert the probabilistic outputs of the final classifier into binary predictions (AM vs. control) by minimizing the absolute distance between the specificity and sensitivity of AM prediction.

The statistical significance of the results was estimated by permutation testing, where we randomly permuted the AM and control labels for each subject in the discovery and replication sets. For each permuted discovery dataset, on the basis of the selected AM biotyping assignment solution and ANN classifier, we classified subjects in each resultant AM biotype from control subjects. We repeated this procedure 200 times and reported the statistical significance of classification accuracy for the replication set.

#### Assessing consistency of AM biotypes between MRI scans

The robustness of the AM biotype scoring was also examined with the second scans of the training subjects. We computed the AUC for each AM biotype against the rest on the basis of AM biotype scores predicted by our model. We then used the number of subjects in a biotype as weights and computed the weighed AUC to evaluate the overall robustness of the AM biotype scoring.

#### Genetic association analysis

The ANN classifier’s outputs for each biotype were treated as quantitative traits in our genetic association analysis. The majority of the HCP subjects are European Americans (EAs, *n* = 550) and African Americans (AAs, *n* = 103), with 86 subjects of other races. We performed association tests for each biotype trait and the quantitative AM trait (the output from the non-differentiated AM-vs-control classifier) using the combined EA and AA samples (*n* = 524 in the discovery set, with 114 from 57 monozygotic twins and 48 from 24 dizygotic twins). We used the Genome-wide Efficient Mixed-Model analysis for Association (GEMMA) [54], a linear mixture model that corrects for correlations among related individuals by a genetic relationship matrix calculated from genome-wide SNPs for all subjects. GEMMA also takes into account population differences in sex and race [55]. We performed quality control steps to the SNPs mapped from previously reported AUD associations. SNPs that were available for <95% of the subjects in the testing or for which the P-value of Hardy–Weinberg equilibrium was <1.0E−07, were excluded. The minor allele frequency (MAF) of each SNP was then calculated within each population in each association test for different traits. SNPs with MAF < 5% in a population were removed from the association tests for the trait. Finally, the selected ANN classifier was applied to the replication sample (*n* = 63, EAs and AAs) to calculate the biotype scores for each subject, allowing us to validate the genetic markers identified from the discovery sample.

## Results

### AM biotypes and the FC features

**Fig. 2:**
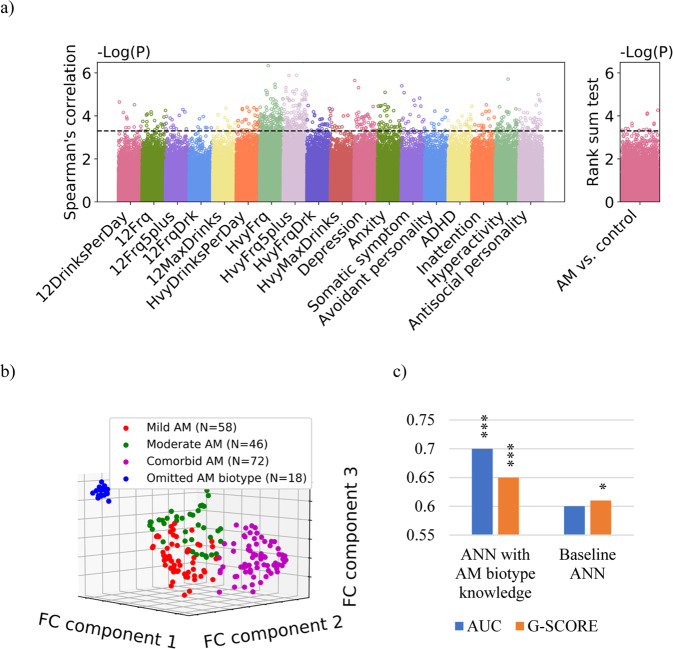
Feature selection, cluster analysis and classification of AM subjects. **a** Manhattan plot showing how significantly each FC feature (a point in each column) is correlated with the alcohol use and clinical metrics (columns on the left; please refer to the section of alcohol use metrics in Table 1 for the full feature names) and differentiates AM from control (column on the right); the higher it is positioned on the plot, the more significant it is. The FC features showing a correlation at *P* < 0.005 (i.e., above the horizontal line) were included in UMAP. **b** K-means clustering results on the basis of the three dimensions revealed by UMAP. **c** Classification performance of the ANN classifier with AM biotype knowledge as compared with baseline ANN (i.e., without biotype knowledge). The implementation of these ANNs is described in Supplementary Methods. G-score = the square root of the product of the sensitivity and specificity of a classifier. *P < 0.05, **P < 0.01, ***P < 0.005 in permutation tests.

After regressing out the effects of age, sex and head motion, Spearman’s rank correlation analysis and Wilcoxon rank-sum test based on the training set identified 18,905, 3505 and 521 FC features, respectively, for *P* < 0.05, 0.005 and 0.0005. Figure 2a shows the Manhattan plots of the distribution of *P* values for the two types of analysis. For each of the three feature sets, UMAP was conducted to reduce the data of AM subjects into lower dimensions (2D, 3D, and 4D). Following the pipeline described in Section 2.5, the 4-cluster AM biotyping solution (Fig. 2b) obtained on the basis of FC features with *P* < 0.0005 in the 3D UMAP space showed the best validation AUC (0.71, *P* = 0.005, permutation test) of the AM classifier. This biotyping solution included a very small group (blue points in Fig. 2b*n* = 18) which we omitted in the subsequent analyses due to lack of power. All remaining three AM biotypes were well replicated on the scan 2 of the training sample (AUC = 0.77, 0.77, 0.71 respectively for biotypes 1, 2, and 3, *P* < 0.05, permutation test, Supplementary Fig. S2). Further, the AM biotypes derived by our approach did not simply recapitulate subtypes derived solely from clinical-symptom measures. As shown in Supplementary Fig. S3, biotyping according to FC features yielded more stable clustering outcomes than that via alcohol use behavior and clinical metrics.

The demographic characteristics were comparable among AM biotypes 1, 2, 3 and the control group (Supplementary Table S5) except for sex. Therefore, with sex as a covariate, we compared the alcohol use and psychiatric metrics between the AM biotypes and between the biotypes and control group. As expected, AM biotypes showed higher scores than the control group in the great majority of alcohol use and psychiatric metrics. Table 1 lists items that showed significant differences among the three AM biotypes (Supplementary Table S6a, b). The three biotypes differed significantly on the frequency of any alcohol use in past 12 months, frequency of drinking 5+ drinks in past 12 months, heaviest frequency of any alcohol use in heaviest 12-month drinking period of participant’s lifetime; and frequency of drinking 5+ drinks during heaviest 12-month drinking period of participant’s lifetime. Specifically, biotype 3 and 2 showed most and least frequent drinking, respectively. Biotype 2 relative to 1 and 3 overall demonstrated higher scores for depression, anxiety, avoidant personality, ADHD, hyperactivity and antisocial personality but only significantly higher for antisocial personality score. Considering the differences in alcohol use and psychiatric metrics, we named AM biotype 1, 2 and 3 each as mild, comorbid, and moderate AM, respectively.

**Table 1:** Comparison of alcohol use and psychiatric features among the three biotypes and controls.

	Mild AM (biotype 1, *n* = 58)	Comorbid AM (biotype 2, *n* = 46)	Moderate AM (biotype 3, *n* = 72)	Control (*n* = 397)	*P*-value^1^
**Alcohol use metrics; mean(SD)**					
Drinks consumed per drinking day in past 12 months (12DrinksPerDay)	2.0 (1.4)	2.5 (1.1)	2.5 (1.3)	1.4 (1.1)	MIA, COA, MOA > CG ^***^
Frequency of any alcohol use in past 12 months (12Frq)	2.4 (1.1)	2.0 (0.8)	2.7 (1.0)	1.6 (0.9)	MIA, MOA > CG ^***^ COA > CG ^**^ MOA > COA ^***^
Frequency of drinking 5+ drinks in past 12 months (12Frq5plus)	1.5 (1.2)	1.7 (1.0)	2.1 (1.1)	0.7 (0.9)	MIA, COA, MOA > CG ^***^ MOA > MIA ^***^
Frequency drunk in past 12 months (12FrqDrk)	1.4 (1.1)	1.5 (0.9)	1.8 (1.1)	0.7 (0.9)	MIA, COA, MOA > CG ^***^
Max drinks consumed in a single day in past 12 months (12MaxDrinks)	2.4 (1.4)	2.8 (1.2)	2.9 (1.1)	1.5 (1.1)	MIA, COA, MOA > CG ^***^
Drinks per day in heaviest 12-month drinking period of participant’s lifetime (HvyDrinksPerDay)	3.3 (1.4)	3.5 (1.3)	3.7 (1.2)	2.2 (1.3)	MIA, COA, MOA > CG ^***^
Frequency of any alcohol use in heaviest 12-month drinking period of participant’s lifetime (HvyFrq)	3.1 (1.2)	2.4 (1.2)	3.3 (1.1)	1.4 (1.3)	MIA, COA, MOA > CG ^***^ MOA > COA ^***^ MIA > COA ^*^
Frequency of drinking 5+ drinks during heaviest 12-month drinking period of participant’s lifetime (HvyFrq5plus)	2.5 (1.2)	2.5 (1.0)	3.2 (0.9)	1.2 (1.2)	MIA, COA, MOA > CG ^***^ MOA > COA ^**^ MOA > MIA ^***^
Frequency drunk in heaviest 12-month drinking period of participant’s lifetime (HvyFrqDrk)	2.7 (1.2)	2.6 (1.2)	3.0 (1.0)	1.4 (1.3)	MIA, COA, MOA > CG ^***^
Lifetime max drinks consumed in single day (HvyMaxDrinks)	3.7 (1.5)	3.7 (1.4)	3.8 (1.3)	2.1 (1.3)	MIA, COA, MOA > CG ^***^
**Psychiatric features; mean(SD)**					
Depression	47.7 (9.9)	54.9 (13.1)	51.4 (11.8)	48.0 (8.3)	COA > MIA, CG ^***^ MOA > CG ^*^
Anxiety	47.0 (9.1)	52.9 (12.5)	50.1 (11.3)	48.2 (9.0)	COA > MIA, CG ^***^
Avoidant personality	47.3 (8.1)	53.2 (12.8)	48.7 (10.1)	49.8 (10.0)	COA > MIA ^*^
ADHD	49.1 (10.9)	54.4 (11.9)	50.0 (10.2)	48.3 (8.8)	COA > CG ^***^ COA > MIA ^*^
Inattention	50.0 (11.5)	54.2 (13.4)	49.6 (9.2)	48.7 (9.1)	COA > CG ^***^
Hyperactivity	48.3 (9.6)	53.5 (9.5)	50.5 (10.6)	48.2 (8.9)	COA > CG ^***^ COA > MIA ^*^
Antisocial personality	48.7 (7.7)	54.1 (10.9)	48.8 (8.2)	47.4 (7.2)	COA, MOA > CG ^***^ COA > MIA, MOA ^***^

*MIA* mild AM biptype, *COA* comorbid AM biotype, *MOA* moderate AM biotype, *CG* control.

^***^P < 0.005, ^**^P < 0.01, ^*^P < 0.05

^1^ With sex as the covariate, two-way ANOVA was used to test whether significant differences (*p* < 0.05) existed among mild, comorbid and moderate AM biotypes and control group. If significant differences were found via two-way ANOVA, with sex as the covariate, Tukey post-hoc tests were conducted to identify pairwise differences in these groups.

**Fig. 3:**
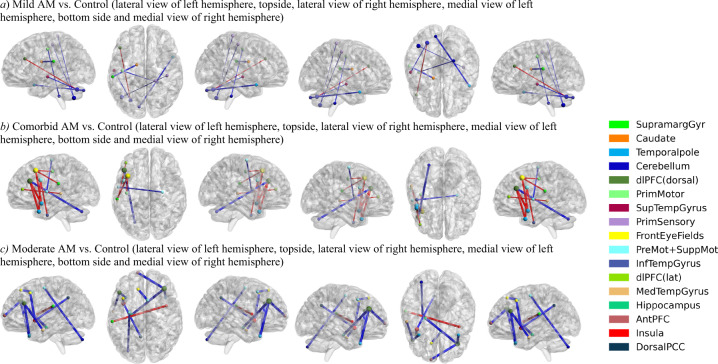
Top FC features differentiating each of the mild AM biotype (a), comorbid AM biotype (b) and moderate AM biotype (c) from control subjects. FC features used in the cluster analysis were considered. All FC features (*n* = 7) that differed significantly (Wilcoxon rank-sum tests, *P* < 0.05, Bonferroni corrected) between mild AM and control groups and the top-10 features that differed most significantly between the other two biotypes from controls were listed. ROIs were colored according to the Brodmann area they belong to, and node size was scaled by its node degree. The edges were colored according to the feature effects: red: AM biotype > control, on average; blue: AM biotype < control, on average. The edge thickness corresponded to the negative-logged *P*-value. Detailed information about FC features in **a**, **b** and **c** can be found in Supplementary Table S7.

We compared the selected 521 FC features between each of the AM biotypes and the control group. Supplementary Table S7 shows the test results and reproducibility of the Wilcoxon rank-sum tests of the FC features that significantly differentiated any AM biotype from control (*P* < 0.05, Bonferroni corrected). Moderate AM identified the greatest number of FC features (*n* = 65), as compared to mild (7) and comorbid (16) AM, that differed significantly from controls. In Fig. 3, we showed the top findings of these FC features. Mild AM vs. control showed higher cerebellar FC with the dorsolateral prefrontal cortex (dlPFC), lower supramarginal gyrus (SMG) FC with the caudate, and lower cerebellar FCs with the temporal pole (TP), superior temporal gyrus (STG), somatosensory and primary motor cortex. Comorbid AM vs. control showed lower dlPFC FC with the cerebellum and higher dlPFC, premotor and supplementary motor cortical FCs with the temporal, including the temporal pole, and parietal cortices. Moderate AM relative to control showed higher insula FC with the SMG, lower premotor and supplementary motor cortical FCs with the temporal pole, medial temporal gyrus and cerebellum, and lower dlPFC FCs with the temporal pole, medial temporal gyrus, and hippocampus. Thus, the three AM biotypes demonstrated FC features that can be distinguished from controls.

### Distinguishing alcohol misuse from control with vs. without biotyping

On the replication set, we compared the baseline ANN model and ANN model with AM biotype knowledge on the basis of the AUC and G-score as shown in Fig. 2c. With AM biotyping, ANN model achieved the highest AUC (0.70, *P* < 0.005) and G-score (0.65, *P* < 0.005), as compared to the results without AM biotyping knowledge (AUC = 0.60, not significant; G-score = 0.61, *P* < 0.05).

### Genetic association analysis

We collected the mild, comorbid, and moderate AM classification scores from the ANN model and used them as the quantitative traits representing these biotypes in genetic analysis. We also extracted the output of the baseline ANN model treating it as the quantitative trait of AM vs. control. Genetic association tests were performed for the four traits first using the 524 training subjects (447 EAs and 77 AAs) in the discovery set, with age, sex and race as covariates, and then the identified SNPs were tested in the replication set (Supplementary Table S8). As Bonferroni correction for all SNPs is highly conservative and would “overcorrect” for SNPs that are not truly independent, we estimated the number of independent SNPs for Bonferroni correction. Specifically, following Duggal et al. [56], we performed blocks of linkage disequilibrium (LD) on the selected SNPs and evaluated the number of independent SNPs by counting 1 SNP per LD block, plus all SNPs outside the blocks.

Table 2 lists the top five SNPs that were identified for each of the four traits. A SNP (rs16930842) in the gene *LINC01414* was significantly associated with the comorbid AM biotype (*P* = 3.52E−05, LD adjusted Bonferroni correction gave *P* < 0.05). This finding was successfully replicated using 63 EAs and AAs combined in the replication set (*P* < 0.05).

**Table 2:** Top SNPs of main effect on different traits (ranked by *P*-value).

Rank	SNP	Chr	Gene	Discovery		Replication	
				MAF	*P*-value^1^	MAF	*P*-value^1^
**Mild AM vs. control**							
1	rs7977619	12	*CACNA1C*	0.073	6.27E-05	0.079	9.39E-01
2	rs151401	4	*SLC39A8*	0.198	1.40E-04	0.23	4.61E-01
3	rs8058681	16	*ZFHX3*	0.193	3.49E-04	0.183	6.63E-01
4	rs13107325	4	*SLC39A8*	0.064	5.00E-04	0.063	6.18E-01
5	rs233807	4	*SLC39A8*	0.194	8.71E-04	0.238	2.28E-01
**Comorbid AM vs. control**							
1	rs16930842^*+^	8	*LINC01414*	0.081	3.52E-05	0.071	1.14E-02
2	rs17717967	14	*OTX2-AS1*	0.079	7.57E-05	0.063	4.10E-01
3	rs6977715	7	*DPP6*	0.237	2.13E-04	0.167	7.47E-01
4	rs59129900	7	*AUTS2*	0.091	3.78E-04	0.079	1.30E-01
5	rs6553691	4	*GALNT7*	0.075	5.04E-04	0.087	2.13E-01
**Moderate AM vs. control**							
1	rs3911063	3	*CADM2*	0.328	5.25E-05	0.31	6.65E-01
2	rs7629375	3	*CADM2*	0.43	2.60E-04	0.397	9.29E-01
3	rs4600827	3	*CADM2*	0.368	5.03E-04	0.357	9.52E-01
4	rs13082138	3	*CADM2*	0.372	5.36E-04	0.357	9.52E-01
5	rs4888559	16	*ZFHX3*	0.483	6.35E-04	0.46	2.71E-01
**AM vs. control**							
1	rs3911063	3	*CADM2*	0.328	1.17E-04	0.31	8.09E-01
2	rs12580201	12	*ACSS3*	0.184	1.35E-04	0.143	6.80E-01
3	rs7188162	16	*FTO*	0.106	3.79E-04	0.079	8.58E-01
4	rs9937234	16	*FTO*	0.116	1.09E-03	0.135	7.83E-02
5	rs12448205	16	*FTO*	0.237	1.19E-03	0.175	7.06E-01

Chr: chromosome, MAF: minor allele frequency.

^*^P < 0.05 on training samples in the discovery set (*n* = 524), LD adjusted Bonferroni corrected.

^+^*P* < 0.05 on samples in the replication set (*n* = 63).

^1^ Significances were tested by Wald test.

## Discussion and conclusion

We employed connectivity markers to group subjects with alcohol misuse (AM). We identified three clusters of AM subjects (biotypes)—mild, comorbid, and moderate—each with a distinct pattern of functional connectivities (FCs) and clinical characteristics including alcohol use metrics that can be differentiated from controls. The comorbid AM biotype also carried potentially unique genetic markers. These findings add significantly to extant effort in characterizing and developing diagnostic biomarkers of alcohol misuse [57–59]. Importantly, with AM subtyping, replication achieved superior AUC and G-score in predicting AM as compared to the analyses without AM biotype differentiation. These results together suggest that reducing the heterogeneity in the FC features among AM subjects by subtyping helps in distinguishing problem drinkers from controls.

FC features are highly non-linear [60, 61]. Linear dimension reduction, such as principal component analysis, did not yield any clustering solutions (data not shown). As a non-linear algorithm, UMAP reduces the dimension of FC features with an iterative process to search for coordinates in a lower dimensional space that preserves the sample proximity. Here, the UMAP dimensions helped K-means and hierarchical clustering of AM biotypes. Thus, the FC features partake in the classification in a non-linear manner and, as a result, it may not be possible to explain how individual connectivity features compose the UMAP dimensions that distinguish AM biotypes. However, by examining which FC features were selected more frequently and prominently by the classifier, we could attempt to understand their roles and relate these findings to those reported in the literature.

With AM biotyping, we identified FC features that distinguished individual AM biotypes from controls. For instance, while demonstrating predominantly lower FCs, mild and moderate AM showed lower supramarginal gyrus (SMG) FCs with the caudate and higher SMG FCs with the insula, respectively, relative to controls. The SMG is a hub of the ventral attention network; a post-hoc explanation is that, while mild alcohol misuse influences the functional integrity of the saliency network, more severe alcohol misuse may lead to compensatory increase in SMG connectivity to support processing of salient stimuli and working memory/executive control, as subserved by the insula and caudate. These findings are broadly consistent with studies implicating the saliency/attention networks in alcohol use severity and relapse [62–64]. Both mild and comorbid AM biotypes showed less drinking severity (relative to moderate AM) and lower dorsolateral prefrontal cortex (dlPFC) FCs with the cerebellum, as compared to controls. An earlier study showed that youth with vs. without a family history of alcoholism showed less response in both the dlPFC and cerebellum during risky decision-making [65]. As impulsivity and sensation seeking represent a critical feature of early alcohol use, one might speculate that disrupted dlPFC cerebellum FCs may characterize this early stage of alcohol misuse and/or dispose individuals to problem drinking. Finally, characterized by higher antisocial scores, comorbid but not mild or moderate AM showed higher dlPFC FCs with the temporal, including the temporal pole, and parietal cortices. The temporal cortex is known for its role in processing social emotions and interaction with the PFC in decision making in social contexts [66]. Inmates with higher psychopathic traits showed altered hemodynamic response in the dlPFC and functional connectivity between temporal, parietal and prefrontal cortices when viewing morally laden interactions [67]. These findings appear to suggest a distinct neural marker of comorbid AM that may not be directly related to alcohol misuse.

Genetic association tests identified a significant SNP (rs16930842) of *LINC01414* for the comorbid AM at a LD adjusted Bonferroni corrected *P*-value (*P* = 3.51E-5), a finding validated in the replication set (*P* = 0.01). *LINC01414* was reported in an early GWAS to be correlated with AUD and antisocial behavior [68], consistent with the current findings.

Several limitations need to be considered for the study. First, the discovery and replication samples were recruited from a single study site; an independent test sample would be needed to eliminate potential site confounds. On the other hand, we did not use the replication set in training the classifiers, thus providing more objective validation as compared with many other AUD classification studies (e.g., [37, 39, 69]). Second, although considered large for imaging studies, the HCP sample size was relatively small for genetic association tests. Thus, we did not perform a GWAS; rather, we extracted SNPs from prior GWAS and identified one significant SNP with LD adjusted Bonferroni correction. Third, although the current data enable analyses of brain circuits, genetics, and behavior at the level of individual subjects [70], the HCP was not a study specifically of alcohol misuse; more alcohol use-related information (e.g., withdrawal symptom severity) would have helped in explaining the differences among mild, comorbid and moderate AM biotypes. Finally, other neural markers such as gray matter volumes and graph-theoretic network measures can also be used as features in our biotyping and classification scheme [71–74]. We intend to pursue these metrics as features in more studies.

## Supplementary information

Zhu et al. Identifying Alcohol Misuse Biotypes from Neural Connectivity Markers and Concurrent Genetic Associations

Table S3 in the supplemental material

Table S8 in the supplemental material

## Data Availability

The anonymized code will be made available to all interested parties upon request.
