# Supplementary material for: Identifying alcohol misuse biotypes from neural connectivity markers and concurrent genetic associations
Source: Transl Psychiatry. 2022 Jun 16;12:253. doi: 10.1038/s41398-022-01983-1 (PMC9203552; doi:10.1038/s41398-022-01983-1)
Supplement: Supplementary file 1 — Zhu et al. Identifying Alcohol Misuse Biotypes from Neural Connectivity Markers and Concurrent Genetic Associations [file 41398_2022_1983_MOESM1_ESM.docx]

**Zhu et al. Identifying Alcohol Misuse Biotypes from Neural Connectivity Markers and Concurrent Genetic Associations**

**SUPPLEMENTARY MATERIALS**

**1. Training Procedure of Artificial Neural Network (ANN)**


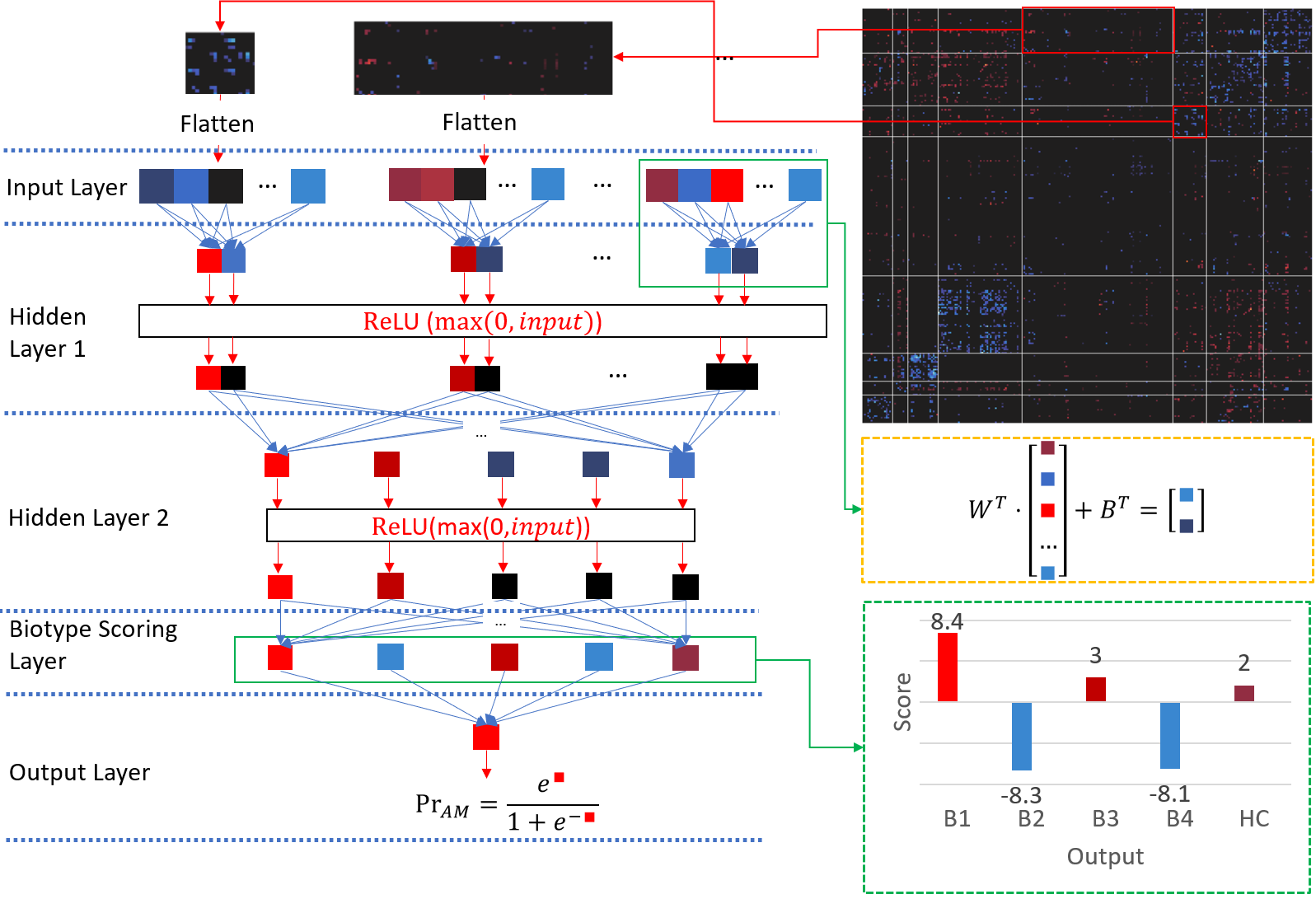


**Figure S1.** *The workflow of the proposed ANN illustrated with a solution of four biotypes. Red, blue and black rectangles mean positive number, negative number, and zero, respectively. Blue arrows represent weighted summation, where matrices W and B are trainable weights. The Rectified Linear Units (ReLUs) in Hidden Layer 1 and 2 output the input directly if it is positive; otherwise, it output zero.*

In the ANN model, the high dimensional whole-brain functional connectivity (FC) matrix was mapped into a low dimensional representation in two steps. In the first step, based on research on alcohol-use predicting FC features ^1^, the whole brain FC matrix was first split into 8 within-network and 28 between-network FC sub-matrices, and FC features in each sub-matrix were mapped into a low-dimensional vector space (i.e., embedding) by calculating linear combinations of FC features and transforming the results with the widely used rectified linear unit (ReLU) in the ANN. In the second step, the low dimensional representation for the whole-brain FC matrix was obtained by calculating ReLU-processed weighted summations of all within- and between-networks’ embeddings. ANN with this local-to-global mapping scheme can learn nonlinear mappings from the whole-brain FC matrix with less trainable ANN parameters, as compared with conventional ANN.

The error of biotype and control scores was measured by the multi-class classification hinge loss, while that of the alcohol misuse (AM) score was evaluated by the *Sigmoid* followed by a *binary* cross-entropy loss (BCEWITHLOGITSLOSS) ^2^. In order to jointly learn the biotype scoring (the output of the Biotype scoring layer) and the AM prediction (the output of the output layer), Adaptive Moment Estimation (Adam) ^3^, a widely used optimization algorithm for training ANN models, was used to minimize the convex combination of the hinge loss and the cross-entropy loss by changing the trainable parameters in the ANN model. AM biotype assignment solution and hyperparameters in this model (the learning rate, the iteration number, and the scalar in the convex combination) need to be adjusted.

To demonstrate that the AM biotyping knowledge could improve ANN’s performance in distinguishing AM subjects from controls, the final evaluation was compared with ANN’s performance in classifying AM from controls without the knowledge of the AM biotyping, in which only the cross-entropy measuring the error of AM prediction was minimized.

**2. Supplementary Tables and Figures**

**Table S1. Demographic characteristics of alcohol misuse (AM)** **and controls.**

| Characteristic | AM (n=250) | Control (n=489) | P-value^1^ |
| --- | --- | --- | --- |
| Age [Years] | 28.3(3.6) | 28.8(3.8) | 9.70E-02 |
| Sex [N(%)] |  |  | 1.63E-03 |
| Men | 132(52.8) | 189(38.7) |  |
| Women | 118(47.2) | 300(61.3) |  |
| Race [N(%)] |  |  | 2.34E-01 |
| African-American (AA) | 28(11.2) | 75(15.3) |  |
| European-American (EA) | 195(78.0) | 355(72.6) |  |
| Other | 27(10.8) | 59(12.1) |  |
| Education [Years] | 15.3(1.6) | 15.1(1.7) | 1.61E-01 |

Numerical values are mean ± SD.

^1^ Discrete variables were tested by the χ^2^-test, whereas numerical variables were tested by the rank-sum test. **Table S2. Alcohol use metrics of the HCP.**

| Description | Variable Name in SSAGA Alcohol |
| --- | --- |
| Drinks consumed per drinking day, past 12 m | 12DrinksPerDay |
| Frequency of any alcohol use, past 12 m | 12Frq |
| Frequency of drinking 5+ drinks, past 12 m | 12Frq5plus |
| Frequency drunk, past 12 m | 12FrqDrk |
| Max drinks consumed in a single day, past 12 m | 12MaxDrinks |
| Drinks per day in heaviest 12-m drinking period of participant's lifetime | HvyDrinksPerDay |
| Frequency of any alcohol use in heaviest 12-m drinking period of participant's lifetime | HvyFrq |
| Frequency of drinking 5+ drinks during heaviest 12-m drinking period of participant's lifetime | HvyFrq5plus |
| Frequency drunk in heaviest 12-m drinking period of participant's lifetime | HvyFrqDrk |
| Lifetime max drinks consumed in single day | HvyMaxDrinks |

**Table S3.** **The alcohol-related genes selected from the NHGRI-EBI GWAS Catalog** ^4^**.**

In the NHGRI-EBI GWAS Catalog, 150 European American (EA)- or African American (AA)-related Single nucleotide polymorphisms (SNPs) in 80 genes were identified as related to the keyword “alcohol use disorder measurement” with P < 5E-8. Then by checking *Genome Reference Consortium Human Build 37 patch release 13* (<https://www.ncbi.nlm.nih.gov/assembly/GCF_000001405.25/>), which shows the positions of SNPs in each of the genes. 78 genes containing 12128 SNPs (genetic variants) were further identified with corresponding data in the genotypes of the HCP database. After conducting quality control, we finally got 3890 SNPs in 75 AUD-related genes. These genetic variants were then included in our genotype association analysis. Table S3 summarizing the selected SNPs can be found in the file *Table_S3_Selected_SNPs*.xls.

**Table S4(a). Demographic characteristics of training, validation, and replication samples.**

| Characteristic | Training (n=591) | Validation (n=74) | Replication  (n=74) | P-value^1^ |
| --- | --- | --- | --- | --- |
| Age [Years] | 28.6(3.7) | 29.1(3.6) | 28.5(4.1) | 5.1E-01 |
| Sex [N(%)] |  |  |  | 3.0E-01 |
| Men | 261(44.2) | 26(35.1) | 34(45.9) |  |
| Women | 330(55.8) | 48(64.9) | 40(54.1) |  |
| Race [N(%)] |  |  |  | 5.1E-01 |
| African-American (AA) | 77(13.0) | 14(18.9) | 12(16.2) |  |
| European-American (EA) | 447(75.6) | 52(70.3) | 51(68.9) |  |
| Other | 67(11.3) | 8(10.8) | 11(14.9) |  |
| Education [Years] | 15.2(1.7) | 15.0(1.8) | 15.5(1.4) | 6.1E-01 |
| Head motion | 639.7(726.1) | 468.0(630.3) | 583.1(708.9) | 4.2E-01 |

Numerical values are mean ± SD.

^1^ Discrete variables were tested by the χ^2^-test, whereas numerical variables were tested by the Kruskal-Wallis H-test.

**Table S4(b). Alcohol use disorder (AUD) and alcohol use features of training, validation, and replication samples.**

| Features | Training (n=591) | Validation (n=74) | Replication  (n=74) | P-value^1^ |
| --- | --- | --- | --- | --- |
| AUD [N(%)] | 78(13.2) | 14(18.9) | 14(18.9) | 2.1E-01 |
| Binge drinking at least once a week in the past 12 months [N(%)] | 79(13.4) | 9(12.2) | 7(9.5) | 6.3E-01 |
| Alcohol use metrics |  | | | |
| Drinks consumed per drinking day in past 12 months (12DrinksPerDay) | 1.7(1.2) | 1.7(1.2) | 1.7(1.2) | 9.4E-01 |
| Frequency of any alcohol use in past 12 months (12Frq) | 1.9(1.1) | 1.9(1.0) | 1.8(1.1) | 2.5E-01 |
| Frequency of drinking 5+ drinks in past 12 months (12Frq5plus) | 1.1(1.1) | 1.1(1.2) | 0.9(1.0) | 6.8E-01 |
| Frequency drunk in past 12 months (12FrqDrk) | 1.0(1.0) | 1.1(1.1) | 1.0(0.9) | 4.8E-01 |
| Max drinks consumed in a single day in past 12 months (12MaxDrinks) | 1.9(1.3) | 1.9(1.2) | 1.9(1.2) | 8.4E-01 |
| Drinks per day in heaviest 12-month drinking period of participant's lifetime (HvyDrinksPerDay) | 2.6(1.4) | 15.8(6.8) | 15.7(5.7) | 3.0E-01 |
| Frequency of any alcohol use in heaviest 12-month drinking period of participant's lifetime (HvyFrq) | 1.9(1.5) | 2.7(1.4) | 2.3(1.4) | 6.2E-01 |
| Frequency of drinking 5+ drinks during heaviest 12-month drinking period of participant's lifetime (HvyFrq5plus) | 1.7(1.4) | 2.1(1.4) | 1.9(1.5) | 8.3E-01 |
| Frequency drunk in heaviest 12-month drinking period of participant's lifetime (HvyFrqDrk) | 1.9(1.4) | 1.8(1.4) | 1.6(1.4) | 7.1E-01 |
| Lifetime max drinks consumed in single day (HvyMaxDrinks) | 2.6(1.5) | 2.0(1.3) | 1.8(1.3) | 5.7E-01 |

^1^ Discrete variables were tested by the χ^2^-test, whereas numerical variables were tested by the Kruskal-Wallis H-test.

^2^ A subject will be diagnosed as AUD if she/he was diagnosed with alcohol dependence (met at least 3 alcohol dependence criteria) or alcohol abuse (met at least 3 alcohol abuse criteria).

**Table S4(c). Psychiatric features of training, validation, and replication samples.**

| Clinical features | Training (n=591) | Validation (n=74) | Replication  (n=74) | P-value^1^ |
| --- | --- | --- | --- | --- |
| Depression | 49.1(9.6) | 52.1(12.0) | 49.9(9.6) | 1.3E-01 |
| Anxiety | 48.8(9.8) | 51.5(10.5) | 50.0(8.9) | 1.7E-01 |
| Somatic symptom | 49.3(9.6) | 52.0(11.0) | 48.2(7.0) | 1.0E-01 |
| Avoidant personality | 49.6(10.1) | 52.1(11.3) | 51.1(8.8) | 2.4E-01 |
| ADHD | 49.2(9.6) | 49.9(9.8) | 49.9(9.5) | 4.6E-01 |
| Inattention | 49.5(9.9) | 49.9(9.4) | 50.0(10.2) | 2.9E-01 |
| Hyperactivity | 49.1(9.4) | 50.0(10.1) | 49.9(9.2) | 7.7E-01 |
| Antisocial personality | 48.4(8.0) | 50.2(10.4) | 49.6(9.3) | 1.6E-01 |

^1^ Variables were tested by the Kruskal-Wallis H-test.


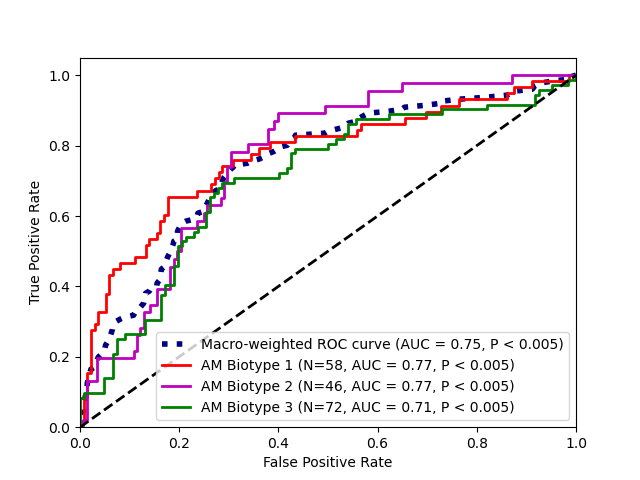


**Figure S2.** *Assessing consistency of alcohol misuse (AM) biotypes between MRI scans. We created classifiers for each of the three biotypes derived from scan 1 rsFMRI data to classify subjects in a biotype from the rest of the AM training subjects based on scan 1 data. We then tested the classifiers on the scan 2 data of these subjects. Permutation tests (200 trials) were performed for each of these classifiers to test statistical significances. All three classifiers had significant AUC with P value < 0.005. The Receiver Operating Characteristic curves (ROC) obtained on scan 2 data are shown in the figure together with AUC. In addition, the macro-weighted ROC curve where the number of subjects in a biotype was used to compute the weighted AUC is also provided to evaluate the overall robustness of the AM biotype scoring.*

1. *Variance ratio criterion calculated for the biotyping solutions obtained, respectively, from resting-state functional magnetic resonance imaging (rsFMRI) and from clinical symptoms.*

1. *Stacked histograms of the P values of rsFMRI features in the Wilcoxon Rank Sum tests with respect to how they are predictive of the resultant biotypes.*

| 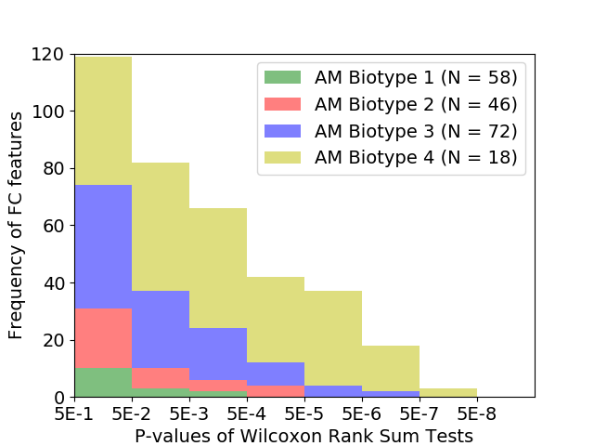 | 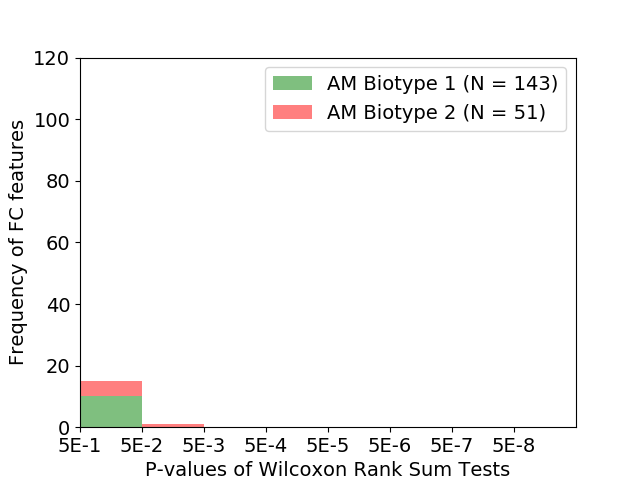 |
| --- | --- |

*rsFMRI-identified biotypes* *Clinical-symptom-identified biotypes*

**Figure S3.** *Following the established method by Drysdale et. al.* ^5^*, we used the variance ratio to measure the stability of the clustering solutions. Biotyping according to rsFMRI features yielded more stable clustering outcomes than that via alcohol use behavior and clinical metrics. (a) The variance ratio criterion was maximized at K=2 for clustering based on the clinical features listed in* ***Figure 2a****. Thus, the 2-cluster solution was used in Wilcoxon Rank Sum tests to see if any neural markers can significantly differentiate the clinical subtypes from healthy controls. The variance ratios for rsFMRI biotypes were much greater as plotted in blue. (b) Stack histograms for the selected 521 rsFMRI features’ P-values in Wilcoxon Rand Sum tests: left: biotypes from rsFMRI; right: biotypes from clinical-symptom measures. P-values were adjusted by the Bonferroni correction method.*

**Table S5. Demographic characteristics of alcohol misuse (AM) biotypes and controls.**

| Characteristic | Mild AM  (Biotype 1, n=58) | Comorbid AM (Biotype 2, n=46) | Moderate AM (Biotype 3, n=72) | Control (n=397) | P-value^1^ |
| --- | --- | --- | --- | --- | --- |
| Age | 28.8(3.9) | 28.2(3.4) | 28.6(3.6) | 28.6(3.8) | 8.4E-01 |
| Sex[N(%)] |  |  |  |  | 6.2E-03 |
| Men | 32(55.2) | 29(63.0) | 35(48.6) | 160(40.3) |  |
| Women | 26(44.8) | 17(37.0) | 37(51.4) | 237(59.7) |  |
| Race[N(%)] |  |  |  |  | 3.1E-01 |
| African-American (AA) | 9(15.5) | 4(8.7) | 3(4.2) | 55(13.9) |  |
| European-American (EA) | 44(75.9) | 36(78.3) | 61(84.7) | 294(74.1) |  |
| Other | 5(8.6) | 6(13.0) | 8(11.1) | 48(12.1) |  |
| Education [Years] | 15.5(1.5) | 15.5(1.6) | 15.5(1.7) | 15.1(1.7) | 8.5E-02 |
| Head motion | 647.3(766.5) | 672.0(721.4) | 613.5(773.3) | 554.9(681.4) | 3.4E-01 |

^1^ Discrete variables were tested by χ^2^-test, whereas numerical variables were tested by the Kruskal-Wallis H test.

**Table S6 (a). Alcohol use disorder (AUD) and alcohol use features of the three alcohol misuse (AM) biotypes.**

| Features | Mild AM  (Biotype 1, n=58) | Comorbid AM (Biotype 2, n=46) | Moderate AM (Biotype 3, n=72) | Control (n=397) | P-value of pairwise comparison^1^ |
| --- | --- | --- | --- | --- | --- |
| AUD^2^[Male] | 12(20.7) | 11(23.9) | 19(26.4) | 0(0.0) |  |
| AUD^2^[Female] | 10(17.2) | 5(10.9) | 14(19.4) | 0(0.0) |  |
| Binge drinking at least once a week in the past 12 months (Male) [%]^***^ | 9(15.5) | 7(15.2) | 17(23.6) | 21(5.3) | MIA, MOA > CG ^***^ |
| Binge drinking at least once a week in the past 12 months (Female) [%]^***^ | 5(8.6) | 2(4.3) | 12(16.7) | 3(0.8) | MIA, MOA > CG ^***^ |
| **Alcohol use metrics** |  | | | | |
| Drinks consumed per drinking day in past 12 months (12DrinksPerDay) ^***^ | 2.0(1.4) | 2.5(1.1) | 2.5(1.3) | 1.4(1.1) | MIA, COA, MOA > CG ^***^ |
| Frequency of any alcohol use in past 12 months (12Frq) ^***^ | 2.4(1.1) | 2.0(0.8) | 2.7(1.0) | 1.6(0.9) | MIA, MOA > CG ^***^  COA > CG ^**^  MOA > COA ^***^ |
| Frequency of drinking 5+ drinks in past 12 months (12Frq5plus) ^***^ | 1.5(1.2) | 1.7(1.0) | 2.1(1.1) | 0.7(0.9) | MIA, COA, MOA > CG ^***^  MOA > MIA ^***^ |
| Frequency drunk in past 12 months (12FrqDrk) ^***^ | 1.4(1.1) | 1.5(0.9) | 1.8(1.1) | 0.7(0.9) | MIA, COA, MOA > CG ^***^ |
| Max drinks consumed in a single day in past 12 months (12MaxDrinks) ^***^ | 2.4(1.4) | 2.8(1.2) | 2.9(1.1) | 1.5(1.1) | MIA, COA, MOA > CG ^***^ |
| Drinks per day in heaviest 12-month drinking period of participant's lifetime (HvyDrinksPerDay) ^***^ | 3.3(1.4) | 3.5(1.3) | 3.7(1.2) | 2.2(1.3) | MIA, COA, MOA > CG ^***^ |
| Frequency of any alcohol use in heaviest 12-month drinking period of participant's lifetime (HvyFrq) ^***^ | 3.1(1.2) | 2.4(1.2) | 3.3(1.1) | 1.4(1.3) | MIA, COA, MOA > CG ^***^  MOA > COA ^***^  MIA > COA ^*^ |
| Frequency of drinking 5+ drinks during heaviest 12-month drinking period of participant's lifetime (HvyFrq5plus) ^***^ | 2.5(1.2) | 2.5(1.0) | 3.2(0.9) | 1.2(1.2) | MIA, COA, MOA > CG ^***^  MOA > COA^**^  MOA > MIA ^***^ |
| Frequency drunk in heaviest 12-month drinking period of participant's lifetime (HvyFrqDrk) ^***^ | 2.7(1.2) | 2.6(1.2) | 3.0(1.0) | 1.4(1.3) | MIA, COA, MOA > CG ^***^ |
| Lifetime max drinks consumed in single day (HvyMaxDrinks) ^***^ | 3.7(1.5) | 3.7(1.4) | 3.8(1.3) | 2.1(1.3) | MIA, COA, MOA > CG ^***^ |

MIA: mild AM biotype, COA: comorbid AM biotype, MOA: moderate AM biotype, CG: control.

^⁎⁎⁎^ P < 0.005, ^⁎⁎^ P < 0.01, ^⁎^ P < 0.05.

^1^ With sex as the covariate, two-way ANOVA was used to test whether significant differences (P < 0.05) existed among mild, comorbid, and moderate AM biotypes and the control group. If significant differences were found via two-way ANOVA, with sex as the covariate, Tukey post-hoc tests were conducted to identify pairwise differences in these groups.

^2^ A subject will be diagnosed as AUD if she/he was diagnosed with alcohol dependence (met at least 3 alcohol dependence criteria) or alcohol abuse (met at least 3 alcohol abuse criteria). The control group is excluded from the statistical comparison of AUD.

**Table S6 (b).** **Psychiatric features of alcohol misuse (AM) biotypes and controls.**

| Clinical features | Mild AM  (Biotype 1, n=58) | Comorbid AM (Biotype 2, n=46) | Moderate AM (Biotype 3, n=72) | Control (n=397) | P-value^1^ |
| --- | --- | --- | --- | --- | --- |
| Depression^⁎⁎⁎^ | 47.7(9.9) | 54.9(13.1) | 51.4(11.8) | 48.0(8.3) | COA > MIA, CG ^***^  MOA > CG ^*^ |
| Anxiety^⁎⁎⁎^ | 47.0(9.1) | 52.9(12.5) | 50.1(11.3) | 48.2(9.0) | COA > MIA, CG ^***^ |
| Somatic symptom | 49.6(10.1) | 51.6(11.4) | 51.5(12.5) | 48.5(8.5) |  |
| Avoidant personality^*^ | 47.3(8.1) | 53.2(12.8) | 48.7(10.1) | 49.8(10.0) | COA > MIA ^*^ |
| ADHD^⁎⁎⁎^ | 49.1(10.9) | 54.4(11.9) | 50.0(10.2) | 48.3(8.8) | COA > CG ^***^  COA > MIA ^*^ |
| Inattention^⁎^ | 50.0(11.5) | 54.2(13.4) | 49.6(9.2) | 48.7(9.1) | COA > CG ^***^ |
| Hyperactivity^⁎⁎⁎^ | 48.3(9.6) | 53.5(9.5) | 50.5(10.6) | 48.2(8.9) | COA > CG ^***^  COA > MIA ^*^ |
| Antisocial personality^⁎⁎⁎^ | 48.7(7.7) | 54.1(10.9) | 48.8(8.2) | 47.4(7.2) | COA, MOA > CG ^***^  COA > MIA, MOA ^***^ |

MIA: mild AM biotype, COA: comorbid AM biotype, MOA: moderate AM biotype, CG: control.

^⁎⁎⁎^ P < 0.005, ^⁎⁎^ P < 0.01, ^⁎^ P < 0.05.

^1^ With sex as the covariate, two-way ANOVA was used to test whether significant differences (p < 0.05) existed among mild, comorbid, and moderate AM biotypes and the control group. If significant differences were found via two-way ANOVA, with sex as the covariate, Tukey post-hoc tests were conducted to identify pairwise differences in these groups.

**Table S7. Top functional connectivities (FCs) significantly differing alcohol misuse (AM) biotype and controls.**

| Rank ^1^ | ROI 1 ^2^ | BA of ROI 1 | ROI 2 ^2^ | BA of ROI 2 | P-value ^3^ | Reproducibility Probability ^4^ |
| --- | --- | --- | --- | --- | --- | --- |
| Mild AM vs. control | | | | | | |
| 1 | 180 | SupramargGyr (40) | 259 | Caudate | MIA<CG^**^ | 0.811 |
| 2 | 52 | Temporalpole (38) | 246 | Cerebellum | MIA<CG^**^ | 0.772 |
| 3 | 146 | dlPFC(dorsal) (9) | 252 | Cerebellum | MIA>CG^*^ | 0.645 |
| 4 | 103 | Cerebellum | 157 | PrimMotor (4) | MIA<CG^*^ | 0.637 |
| 5 | 196 | SupTempGyrus (22) | 252 | Cerebellum | MIA<CG^*^ | 0.611 |
| 6 | 32 | PrimSensory (1) | 245 | Cerebellum | MIA<CG^*^ | 0.529 |
| 7 | 38 | PrimSensory (1) | 245 | Cerebellum | MIA<CG^*^ | 0.519 |
| Comorbid AM vs. control | | | | | | |
| 1 | 146 | dlPFC(dorsal) (9) | 252 | Cerebellum | COA<CG^***^ | 0.915 |
| 2 | 146 | dlPFC(dorsal) (9) | 186 | Temporalpole (38) | COA>CG^***^ | 0.934 |
| 3 | 148 | FrontEyeFields (8) | 187 | Temporalpole (38) | COA>CG^***^ | 0.893 |
| 4 | 146 | dlPFC(dorsal) (9) | 187 | Temporalpole (38) | COA>CG^***^ | 0.873 |
| 5 | 156 | PreMot+SuppMot (6) | 186 | Temporalpole (38) | COA>CG^**^ | 0.845 |
| 6 | 156 | PreMot+SuppMot (6) | 193 | InfTempGyrus (20) | COA>CG^**^ | 0.776 |
| 7 | 25 | PreMot+SuppMot (6) | 162 | PreMot+SuppMot (6) | COA<CG^**^ | 0.777 |
| 8 | 148 | FrontEyeFields (8) | 180 | SupramargGyr (40) | COA>CG^**^ | 0.813 |
| 9 | 153 | dlPFC(lat) (46) | 190 | MedTempGyrus (21) | COA>CG^*^ | 0.741 |
| 10 | 148 | FrontEyeFields (8) | 157 | PrimMotor (4) | COA>CG^*^ | 0.724 |
| Moderate AM vs. control | | | | | | |
| 1 | 10 | dlPFC(dorsal) (9) | 51 | Temporalpole (38) | MOA<CG^***^ | 0.988 |
| 2 | 27 | PreMot+SuppMot (6) | 147 | FrontEyeFields (8) | MOA<CG^***^ | 0.996 |
| 3 | 10 | dlPFC(dorsal) (9) | 230 | Hippocampus | MOA<CG^***^ | 0.983 |
| 4 | 10 | dlPFC(dorsal) (9) | 140 | AntPFC (10) | MOA<CG^***^ | 0.97 |
| 5 | 146 | dlPFC(dorsal) (9) | 185 | Temporalpole (38) | MOA<CG^***^ | 0.947 |
| 6 | 148 | FrontEyeFields (8) | 168 | Insula (13) | MOA<CG^***^ | 0.964 |
| 7 | 146 | dlPFC(dorsal) (9) | 189 | MedTempGyrus (21) | MOA<CG^***^ | 0.889 |
| 8 | 34 | Insula (13) | 180 | SupramargGyr (40) | MOA>CG^***^ | 0.897 |
| 9 | 165 | PreMot+SuppMot (6) | 241 | Cerebellum | MOA<CG^***^ | 0.886 |
| 10 | 168 | Insula (13) | 224 | DorsalPCC (31) | MOA<CG^***^ | 0.913 |

ROI: region of interest, BA: Brodmann area, MIA: mild AM biotype, COA: comorbid AM biotype, MOA: moderate AM biotype, CG: control.

⁎⁎⁎ P < 0.0005, ⁎⁎ P < 0.005, ⁎ P < 0.05

^1^ we listed all of the 7 FC features that differed significantly between mild AM and control groups and the top 10 features that each differed most significantly between comorbid and moderate AM and controls. The full version of this table can be found in Supplementary Table S8.

^2^ The ID of ROI ranged from 0 to 267.

^3^ Wilcoxon rank-sum tests were used to examine the FC differences between AM biotypes and controls

^4^ The reproducibility probability (RP)^6^ is an indicator measuring the reproducibility of the statistical significance of the wilcoxon rank-sum test.

**Table S8.** **(Full table of Table S7) Functional connectivities (FCs) significantly differing each alcohol misuse (AM) biotype from control subjects.**

FC features significantly differentiating each of AM biotypes from control subjects could be found in the file *Table_S8*.xls.

**Table S9. Demographic characteristics of alcohol misuse (AM) biotypes and controls [African-American (AA) and European-American (EA)].**

| Characteristic | Mild AM  (Biotype 1, n=53) | Comorbid AM (Biotype 2, n=40) | Moderate AM (Biotype 3, n=64) | Control (n=349) | P-value^1^ |
| --- | --- | --- | --- | --- | --- |
| Age | 28.8(3.9) | 28.5(3.3) | 28.9(3.5) | 28.9(3.6) | 9.0E-01 |
| Sex[N(%)] |  |  |  |  | 5.5E-03 |
| Men | 30(56.6) | 26(65.0) | 31(48.4) | 141(40.4) |  |
| Women | 23(43.4) | 14(35.0) | 33(51.6) | 208(59.6) |  |
| Race[N(%)] |  |  |  |  | 3.1E-01 |
| AA | 9(17.0) | 4(10.0) | 3(4.7) | 55(15.8) |  |
| EA | 44(83.0) | 36(90.0) | 61(95.3) | 294(84.2) |  |
| Education [Years] | 15.5(1.6) | 15.3(1.7) | 15.5(1.7) | 15.1(1.7) | 1.0E-01 |
| Head motion | 662.5(775.2) | 727.5(754.2) | 637.6(804.5) | 576.8(696.5) | 3.6E-01 |

^1^ Discrete variables were tested by χ^2^-test, whereas numerical variables were tested by the Kruskal-Wallis H test.

**REFERENCES**

1 Fede SJ, Grodin EN, Dean SF, Diazgranados N, Momenan R. Resting state connectivity best predicts alcohol use severity in moderate to heavy alcohol users. Neuroimage: clinical 2019; 22: 101782.

2 Paszke A, Gross S, Massa F, Lerer A, Bradbury J, Chanan G et al. PyTorch: An Imperative Style, High-Performance Deep Learning Library. In: Advances in Neural Information Processing Systems 32. Curran Associates, Inc., 2019, pp 8024–8035.

3 Kingma DP, Ba JL. Adam: A method for stochastic optimization. In: 3rd International Conference on Learning Representations, ICLR 2015 - Conference Track Proceedings. 2015, pp 1–15.

4 Buniello A, MacArthur JAL, Cerezo M, Harris LW, Hayhurst J, Malangone C et al. The NHGRI-EBI GWAS Catalog of published genome-wide association studies, targeted arrays and summary statistics 2019. Nucleic Acids Res 2019; 47: D1005--D1012.

5 Drysdale AT, Grosenick L, Downar J, Dunlop K, Mansouri F, Meng Y et al. Resting-state connectivity biomarkers define neurophysiological subtypes of depression. Nat Med 2017; 23: 28–38.

6 De Capitani L, De Martini D. Reproducibility probability estimation and testing for the Wilcoxon rank-sum test. Journal of Statistical Computation and Simulation 2015; 85: 468–493.
